# Policies to Reduce Lead Exposure: Lessons from Buffalo and Rochester

**DOI:** 10.3390/ijerph15102197

**Published:** 2018-10-09

**Authors:** Sam Magavern

**Affiliations:** Executive Director, Partnership for the Public Good, Buffalo, NY 14203, USA; sam@ppgbuffalo.org; Tel.: +716-852-4196

**Keywords:** lead, policy, local

## Abstract

Lead exposure remains a major issue in cities, such as Buffalo and Rochester, with concentrated, segregated poverty and old, deteriorated housing stock. Exploring and comparing local policies and programs in these two cities, the author suggests that increasing the number of proactive housing inspections in high-risk areas and forming a single-purpose non-profit group dedicated to lead education and advocacy are two valuable interventions. He recommends additional policy steps, such as more stringent inspection standards; state adoption of the Environmental Protection Agency’s Renovation and Repair Program; the lowering of state elevated blood level thresholds; a focus on in-person, interactive education by community health workers; and more vigorous enforcement of testing requirements among physicians.

## 1. Introduction

Blood lead levels in children have fallen dramatically across the United States in recent decades, but lead exposure remains a significant public health problem, particularly in cities, like Buffalo, New York, where concentrated poverty is combined with old, deteriorated housing stock. The overlap of lead poisoning with racial and economic segregation makes it an important issue of environmental justice. This article discusses how local governments have responded to elevated blood lead levels in Buffalo, with comparisons to efforts in a similar city—Rochester, New York. It describes policies and programs regarding housing inspections, intervention thresholds, renovations, testing, and education, and includes recommendations for further state and local government action.

## 2. Discussion

### 2.1. History and Demographics

Buffalo is a city that boomed from roughly 1825 to 1960 due to its strategic location at the terminus of the Erie Canal on Lake Erie. The shipping of grain, timber, iron ore, and other commodities through Buffalo, combined with cheap hydro-electricity from Niagara Falls, made Buffalo a major manufacturing center that drew waves of immigrants. Starting in the 1970s, the region lost many thousands of manufacturing jobs, and its population began to decline [1]. The Buffalo-Niagara metropolitan region went from nearly 1.4 million residents to roughly 1.1 million [1]. The city of Buffalo itself shrank from almost 600,000 residents in 1950 to approximately 260,000 in 2010 [1].

Even as the region’s population shrank, suburbanization continued at a rapid pace, with wealthier residents, mostly white, increasingly choosing to live outside the city. Today, while the poverty rate for the metropolitan region hovers around the national average, at approximately 15 percent, the poverty rate in the city of Buffalo stands at over 30 percent—one of the highest rates in the nation [2]. People of color are heavily concentrated in certain urban neighborhoods, and the region ranks in the top ten for both racial [3] (p. 45). and economic segregation [4]. The urban housing stock is among the nation’s oldest, with 93 percent built before 1980 [5] (p. 9).

Rochester is a nearby city with a similar poverty rate and similarly old housing stock, making it an apt city for comparison with Buffalo. Rochester is located in Monroe County, while Buffalo is located in Erie County. Monroe County’s population is 81 percent that of Erie County, but in 2016, its number of new cases of children with elevated blood lead levels (EBLLs) of 10 μg/dL or higher was 67 percent that of Erie County [5] (p. 54) [6]. There are many possible explanations for the difference. Erie County may test more high-risk children and so identify more cases (Erie County’s testing rates are consistently higher than Monroe County’s) [5] (p. 16). Erie may have more small, wood-frame residential buildings or worse housing conditions. It may have more members of vulnerable populations (for example, Erie County resettles more refugees than Monroe County) [7]. Finally, some of Rochester/Monroe’s policies and programs may be better funded or more efficient than those in Buffalo/Erie.

### 2.2. Lead Exposure in Buffalo: Scope and Causes

The housing stock in Buffalo is dominated to an unusual extent by small, painted wood-frame buildings: Single-family homes, duplexes, and triples. Compared to other cities, there are few apartment buildings and few buildings made with brick, stone, or stucco. Years of depopulation, segregation, and disinvestment have left Buffalo with one of the most deteriorated housing inventories in the nation [5] (pp. 7–9).

The combination of concentrated poverty with an old, wood-framed housing stock is a recipe for lead exposure, and Buffalo suffers from some of the highest rates of EBLLs in the nation. Other parts of upstate New York experience similar dynamics. In fact, if one excludes New York City, then New York State has some of the highest blood lead levels in the United States. According to the Centers for Disease Control and Prevention, in 2016, 5.6 percent of children tested in New York State outside of New York City had an EBLL equal to or higher than 5 μg/dL [8].

Despite accounting for only 29 percent of Erie County’s population, Buffalo—due to its poverty and old housing stock—accounts for almost all of its lead cases. In 2017, the County identified 466 new cases of children with lead levels between 5 and 9 μg/dL and 290 children with levels of 10 μg/dL or higher [5]. Of the 290 children with levels over 10 micrograms per deciliter in 2018, 49 had levels between 15 and 19, and 55 had levels of 20 or higher, and of those 290 children, 278 lived in the city [5] (p. 54). Within the city, a large majority of the children lived in impoverished and heavily minority neighborhoods [5] (p. 22). It has been estimated that children from Buffalo’s neighborhoods of color are twelve times more likely than children from predominantly white neighborhoods to test with elevated blood levels [3] (p. 43). In 2017, Reuters reported that in four Buffalo zip codes, 40 percent of children tested between 2006 and 2014 had high lead levels, causing many to wonder why the situation in Buffalo had not received the same publicity as Flint, Michigan, where the rates of exposure were substantially lower [9].

Most of the Buffalo children with elevated levels live in duplexes or single-family homes. Of the 2578 children with lead levels over 15 μg/dL between 2008 and 2016, 1948 (76 percent) lived in one, two, or three family homes, while only 244 lived in apartments or multiple residences [5] (p. 55). Roughly 80 percent of the EBLL referrals come from rental housing, as opposed to owner occupied [5] (p. 19). Rather than being concentrated among a few large companies, the rental housing where exposure occurs is owned by a large number of different landlords. Of the 1383 owners of properties with EBLL referrals, 1247 had only one referral [5] (p. 21).

In examining the housing where children are exposed, County sanitarians have found that the source of the lead is nearly always paint—most often dust and chips from windows, doors, siding, and porches; and they have noted that—consistent with research on other regions—cases rise in the summer, when windows get opened and closed, lead dust blows in from exteriors through windows, children play on porches and in yards, and lead dust from porches and soil is tracked into houses [5] (p. 35) [10].

In comparing Erie County with other counties in upstate New York, two things stand out. Erie County has the highest testing rate; and it has the highest percentage of EBLLs among children tested (over 20 percent of children tested in 2015 had a level over 10 μg/dL) [5] (pp. 16–17). These two facts may be related: Erie may test more high-risk children and so discover lead exposure at a higher rate. At any rate, it is clear that Erie County faces a difficult challenge: Despite increased efforts by both the City and County in recent years, EBLLs have not dropped; new cases of EBLLs of 10 μg/dL or higher remained fairly constant at roughly 280 from 2013 to 2017 [5] (p. 7).

### 2.3. Local Responses to Lead Exposure: Policies and Programs

How does a community go about preventing lead exposure in its children? Typical responses include testing as many children as possible; educating residents, property owners, and contractors about lead-safe practices; proactively inspecting housing for lead hazards; regulating housing renovation work; and promptly remediating lead hazards once identified. We now examine some of the efforts undertaken in Buffalo, how they compare with those in Rochester, and how they might be modified and supplemented in the future.

Federal and state laws, regulations, and programs regarding lead are critically important, but in this article we will focus mainly on city and county governments. Lead exposure is a health problem caused mainly—in Buffalo—by poor housing conditions in old homes with lead paint. In Buffalo, housing conditions are mainly in the purview of the City and its Permits and Inspections Department, whereas health is mainly in the purview of the County and its Health Department. Both governments, thus, have critical roles to play, and coordination between them is essential.

### 2.4. Proactive Inspections

As we have seen, lead exposure in Buffalo comes mainly from deteriorated paint in residential housing. A key prevention tool, therefore, is proactive inspection of housing units for deteriorated lead paint. Currently, the City requires buildings with three units or more to obtain a certificate of occupancy, which includes a City inspection for code compliance at the time of the initial application and then every three years for renewal [11]. Unfortunately, no certificate is required for buildings with one or two units, which is where over three fourths of EBLL cases occur [5] (p. 55). In Rochester, by contrast, a certificate of occupancy is required for one and two unit buildings if they are not owner occupied [12].

Another tool Buffalo uses to reach smaller buildings is its Rental Registration law, which requires the owners of non-owner-occupied singles and duplexes to register with the City. Buffalo has already amended its law to require that owners of properties built before 1978 certify that they are aware that their properties may contain lead and that they must use lead-safe renovation methods, and to require owners to attest to the City that their tenants have received and signed a lead hazard warning [13]. Buffalo could amend its law to include owner-occupied buildings and require that all owners, in order to register, must pass an inspection for deteriorated paint upon their initial application and every three years thereafter [5] (p. 29).

New York State offers a way to prioritize proactive inspections by need. The State Department of Health authorizes counties to declare high-risk neighborhoods, based on EBLL data; the county’s Health Department then makes its way through those neighborhoods, doing inspections and lead testing, and issuing orders for repairs where needed. Between 2007 and 2015, Erie County used this program to inspect proactively over 7500 units, of which 4846 had exterior lead hazards [5] (p. 11). In addition to these County efforts, the City of Buffalo inspected thousands of units; for example, in 2016 and 2017, the City inspected 5000 residences in high-risk zip codes [5] (p. 12). Rochester/Monroe appears to have reached more units, however. It inspected 43,000 units in high-risk areas between 2006 and 2017, suggesting that Rochester/Monroe was using more inspectors and/or using them more efficiently than Buffalo and Erie County [5] (p. 92).

Another way to increase the impact of proactive inspections is to make them more stringent and to access more interiors. In Rochester, when the high-risk neighborhoods are inspected, if no deteriorated paint or bare soil is found, the property must still pass an interior “dust-wipe” inspection [5] (p. 91). Of the 43,000 units inspected in eleven years, 88 percent were given the dust-wipe test, of which 90 percent passed upon the first test and 98 percent were cleared upon subsequent testing [5] (p. 92). These results suggest that dust-wipe testing successfully identifies lead hazards not flagged by visual inspections and thus leads to more remediation.

Counties have another way to prioritize and implement proactive inspections. In many counties, the social services department assists certain recipients of public assistance with rental payments and/or security deposits. In cases where the county is playing such a role, it can condition its payment to the landlord upon a pre-move-in inspection of the premises to identify health hazards, such as deteriorated paint. If the county is paying or guaranteeing the security deposit, such an inspection can also save the county money by preventing owners from making false claims of damages when the tenant moves out. Non-profit social service agencies that place refugees, ex-offenders, or other vulnerable populations into housing can adopt similar policies to prevent the placement of clients in unsafe housing.

### 2.5. Renovations: Regulating and Educating

Renovations are an important source of lead exposure. In 2008, New York State’s Department of Health reviewed the records of 972 children with EBLLs over 20 μg/dL from 2006–2007 and estimated that 139 (14 percent) of the cases stemmed from remodeling or renovation activities [14].

Under regulations of the federal Environmental Protection Agency, rental property owners or professional contractors who do any work that disturbs paint are subject to the EPA’s Renovation, Repair and Painting Rule (RRP) and must be trained and certified. Too often, however, renovation work is done by owners or contractors who are not RRP trained or who fail to follow the proper lead-safe procedures.

One response is more vigorous enforcement of RRP. The EPA brought only 123 RRP enforcement actions in 2016, including only three in New York State [5] (p. 35). Fourteen states, not including New York, have adopted the RRP Program and do their own training, certification, and enforcement. New York could do the same and then choose to enforce the regulations more vigorously with—for example—random audit inspections of work-sites to make sure that all the workers are properly trained and observing lead-safe practices. A recent report by the Health Impact Project estimated that rigorous enforcement of RRP would protect 211,000 children in the United States and return $3.10 in benefits for every dollar invested [15].

Erie County and the City of Buffalo could also take more action on renovations. For example, to obtain a building permit for work that involves disturbing paint, a contractor in Buffalo currently checks a box attesting to RRP certification, but does not need to provide proof of it (i.e., a copy of the certificate). Both the City and County could also pass legislation on lead-safe work practices and enforce it with inspections and fines [5] (p. 37).

A few well-publicized cases of enforcement would make a significant impact on owners and contractors. In addition, it is important to educate them on the risk that unsafe renovations pose to themselves, their employees, and their tenants, and the potential for legal liability that those risks create.

A question for further research is whether to prioritize full removal of lead paint or interim controls. This will require revisiting the data over time to see how common it is for a property to pass inspection after interim controls are done, but then become the site of additional lead exposure as the paint begins to deteriorate again.

### 2.6. Thresholds

Local and state governments are revisiting the question of what EBLL thresholds should trigger various governmental responses. As research has revealed the negative impacts of low levels of lead exposure, the CDC has lowered its level of concern or “reference value” from 30 μg/dL (1970) to 25 (1985) to 10 (1991) to 5 (2012). Today, the CDC recommends at 5 μg/dL an “environmental assessment of detailed history to identify potential sources of lead exposure.” At 10 μg/dL it recommends an “environmental investigation including home visit,” and at 20 μg/dL it recommends “environmental investigation of home and lead hazard reduction [16].”

Under current New York State laws and regulations, intervention by local health departments is triggered by an “elevated lead level,” which is defined as 10 μg/dL or greater [17]. However, environmental management is triggered only by a level of 15 μg/dL or greater [18]. In February 2016, Erie County began treating 5 μg/dL as its level of concern, hiring a full-time nurse to provide medical case management to children in the 5 to 9 μg/dL range and providing environmental management for levels of 10 μg/dL and above [5] (pp. 11–12). Erie County’s policy is now more expansive than that of Monroe County, which in 2013, lowered its threshold of concern from 10 to 8 μg/dL [19]. If the State of New York were to follow the lead of Erie County and adjust its action levels downward, and then provide increased funding, many more children would receive case management, and many more homes would be remediated.

### 2.7. Testing

Testing may not seem like a prevention strategy; after all, if a child tests with an EBLL, one might argue, the damage has already been done. In fact, however, testing can prevent children with mildly elevated levels from reaching highly elevated levels by identifying and removing the sources of exposure. In addition to helping the child who is tested, the remediation of the source can also help prevent other children from being exposed to it.

Erie County currently boasts one of the highest testing rates in the State, with 61 percent of children born in 2012 tested twice by age 3, a higher rate than in Monroe County (50 percent) [5] (p. v). Further research is needed to learn why 39 percent of children in Erie County and 50 percent of children in Monroe County were not tested twice. Some may be suburban children whose parents or doctors do not perceive a risk because they know the child lives in post-1978 housing. Some may be highly vulnerable children in poverty who are not receiving well-child check-ups. Some may be children who were tested, but, because they did not show elevated levels, their doctor did not bother to report the results to the state. Some may be children who were referred by their doctors for blood draws at separate facilities, but whose parents failed to complete the process.

Research into the causes for failure to test will help determine the solutions. Certain avenues appear promising, however. State law already requires primary care physicians to test children at the ages of one and two. Counties, such as Erie and Monroe, could emulate the Philadelphia Health Department, which monitors the screening rates of physicians and sends them messages identifying children whose test results have not been received [5] (p. 15).

### 2.8. Education

In Buffalo, many government entities and non-profits have been involved in lead poisoning prevention education. In 2016, for example, the County, City, and Buffalo School District partnered with the Community Foundation for Greater Buffalo on a Wipe Out Lead campaign that included 28 billboards, mostly in highly impacted neighborhoods, and similar messaging on 120 buses [20]. The billboard showed a picture of a baby and asked, “Is your baby sucking on poisonous lead?” It then stated that “Homes built before 1978 likely contain lead paint” and that “lead is a poison.” It instructed Buffalo residents to call the City’s all-purpose hotline, 311, to “report lead concerns.” In addition, the School District and City combined to do a direct mailing to all 18,000 public school families [5] (p. 43). The City has not reported an increase in 311 calls regarding lead based on the campaign, although it may have led to residents taking other preventive actions.

It may be wise to supplement such campaigns with in-person and interactive communication targeted directly to vulnerable residents: People living in old rental housing in high-risk neighborhoods. Local governments and non-profits should consider hiring community health workers—people of and from the impacted neighborhoods—to visit homes, schools, places of worship, block clubs, and neighborhood events to talk in quite specific terms about prevention: How to clean up dust that may contain lead; how to make lead-safe repairs; and how to get landlords to make lead-safe repairs. The education should focus on the key sources of lead exposure in Buffalo: Deteriorated paint and lead dust from “friction surfaces” (windows, doors, porches, etc.) and dust created from renovation.

One thing Buffalo lacks is the presence of a funded, single-purpose organization, like Rochester’s Coalition to Prevent Lead Poisoning: A non-profit that has done lead education and advocacy since 2000. Establishing such an organization in Buffalo would add power to existing efforts. The Community Foundation for Greater Buffalo has convened a Lead Poisoning Prevention Coalition with many stakeholders, which could serve as the springboard to a dedicated non-profit organization.

## 3. Conclusions: The Impacts of Policy and Programs

It is hard to assess which local policy and program measures have had the most impact in preventing lead exposure, but Rochester’s history offers some illuminating clues. In 1997, the prevalence rate of tested children with levels of 10 μg/dL or higher was almost 14 percent in Monroe County, roughly twice the rate in the rest of Upstate New York and in the nation. The Monroe County rate fell sharply from 1997 to 2011 and continued to fall steadily until, by 2011, it was below 2 percent and very close to the rate in Upstate New York and in the nation [19] (p. 260). The number of new cases of children with levels of 10 μg/dL or higher reached an all-time low of 139 children in 2013. (It rose to 206 in 2015 [21] and dipped back to 191 in 2016) [6].

What explains the rapid progress Monroe County made? Almost certainly, an important factor was the amount of funding Monroe County obtained and devoted to lead prevention, and the amount of staff it was able to dedicate to its efforts. In the late 1990s, when Monroe made the most rapid progress, it had its highest number of public health staff devoted to lead—the number rose to roughly 20 in 1994 before shrinking to ten by 2005 [19] (p. 261).

While it is impossible to know the precise impact of each intervention, Buffalo and other cities may wish to take particular note of two aspects of Rochester’s efforts. First, Rochester has done an impressive number of proactive inspections, particularly in high-risk areas. Since 2005, this activity has been aided by laws that require dust wipes in high-risk areas and that require lead hazard inspections every three years for a certificate of occupancy. In contrast to Buffalo, Rochester’s certificate of occupancy is required for one and two unit rentals, if they are not owner occupied. Second, Rochester benefits from having a non-profit organization dedicated exclusively to lead education and advocacy. Of course, Rochester is still grappling with significant lead exposure, and it, too, can learn lessons from Buffalo and other cities—for example, how to achieve a higher testing rate. More sharing of research, policies, programs, and results from cities and counties around the nation can help each region to further its progress in reducing lead exposure.

In Buffalo, Rochester, and similar cities, lead exposure occurs most often in high-poverty urban neighborhoods, where, due to residential segregation and racial economic disparities, people of color are heavily concentrated. Thus, lead poisoning prevention is an imperative for both public health and equal justice. The examples cited in this commentary show how directly public policies can reduce exposure; by highlighting both the health and justice impacts, residents can help to generate the political will necessary to enact those policies.
